# Residual Levels of Mercury, Cadmium, Lead and Arsenic in Some Commercially Key Species from Italian Coasts (Adriatic Sea): Focus on Human Health

**DOI:** 10.3390/toxics10050223

**Published:** 2022-04-28

**Authors:** Grazia Barone, Arianna Storelli, Rita Garofalo, Rosanna Mallamaci, Maria Maddalena Storelli

**Affiliations:** Biosciences, Biotechnologies and Biopharmaceutical Department, University of Bari “Aldo Moro”, 70010 Bari, Italy; grazia.barone@uniba.it (G.B.); arianna.storelli@uniba.it (A.S.); rita.garofalo@uniba.it (R.G.); rosanna.mallamaci@uniba.it (R.M.)

**Keywords:** marine fish, metal(loid)s, dietary exposure, risk assessment

## Abstract

This study provides information on the potential human health risk of Hg, Cd, Pb and As exposure from consumption of two fish species (*Umbrina cirrosa* and *Sciaena umbra*) in the general population and in high-level fish consumers. The concentrations did not show significant differences between the two species, and no fish length element level–body-length relationship was observed, except for Hg. The average metal(loid) levels, irrespective of species, varied in the following ranges: Hg = 0.18–0.19, Cd = 0.07–0.10, Pb = 0.10–0.12, As = 0.59–0.69 μg g^−1^ w.w. The concentrations remained below the maximum permissible limits (MPLs) for human consumption, except for Cd. The estimated intakes of Hg, Cd and Pb in both consumption scenarios were lower than the respective PTWI/PTMIs, as well as those of inorganic As, which were even lower than the BMDL_01_. The non-carcinogenic risk (THQ) did not reveal any concerns, except for Hg. The lifetime health cancer risk (ILCR) suggested hazard exclusively from Cd, although for high-level fish consumers, even the ILCR of inorganic As was, in some cases, above the acceptable range. Continuous monitoring of metal(loid) levels in these fish is strongly recommended because the results demonstrate the occurrence of potential health risks, especially in high-level fish consumers, due to the presence of Hg and Cd.

## 1. Introduction

In the recent years, world consumption of fish has strongly increased as result of a more widespread and in-depth knowledge of the benefits of fish for human health [1]. Its consumption accounts for around 17% of the total animal protein consumed by the human population worldwide [2], providing essential minerals and vitamins. In addition, fish and seafood are excellent sources of omega-3 (n − 3) long-chain polyunsaturated fatty acids (LC-PUFA), particularly eicosapentaenoic (EPA: 20:5n − 3) and docosahexaenoic (DHA: 22:6n − 3) acids, well-known for advantageous effects in a range of human pathologies. Epidemiological and clinical evidence suggests that consumption of long-chain polyunsaturated omega-3 fatty acids reduces cholesterol levels and prevents most cardiovascular, neurodegenerative and proinflammatory disorders [3]. For these its exceptional nutritional properties and therapeutic benefits, fish constitutes one of the more healthful foods. Therefore, many public health authorities recommend eating fish at least twice per week [4]. Despite the importance of fish in a well-balanced diet, it should not be forgotten that its consumption is the major route of human exposure to numerous chemical contaminants [5]. Trace metals, due to their toxic potentials, long persistence in nature, possible bioaccumulation in living organisms and biomagnification along food chains, are pollutants of great concern. These chemicals released from natural and anthropogenic sources can reach the marine environment and concentrate in organisms with inevitable transfer to man, who consumes them. Human exposure to mercury (Hg), cadmium (Cd), lead (Pb) and arsenic (As) elements are among the list of top ten chemicals of major public health concern [6] and can result in several acute and chronic adverse effects. For example, a high intake of Hg could cause neurotoxicity, teratogenicity, nephrotoxicity and immunotoxicity. Pb and Cd can produce alterations in physiological functions of the body and are associated with many diseases damaging the liver, kidney and skeletal system [7]. Exposure to As through contaminated food can also lead to serious health effects, including cancer, melanosis, lung disease, hypertension and ischaemic heart disease [7]. Considering the harmful consequences that these chemicals have on human health, many scientific reports about the levels of toxic metals in a wide variety of fish species have been reported all over the world [8,9,10,11,12]. In Italy, where the annual *per capita* consumption of fish is one of the highest within EU countries, at 31.02 kg [13], the quantification of trace metals and the characterization of human exposure risks arising from fish consumption has been explicitly addressed in many studies [14,15,16,17,18]. Despite this and to our best knowledge, there have been no reports on metal levels in fish belonging to the Sciaenidae family, such as *Umbrina cirrosa* (shi drum) and *Sciaena umbra* (brown meagre) from the Italian coasts. This study represents an early investigation on metal(loid) content (Hg, Cd, Pb and As) in the edible part of these two fish species of high consumption and commercial interest throughout the Mediterranean area [19]. The assessment of the potential human health risks using parameters viz estimated intake (EI), target hazard quotient (THQ), hazard index (HI), incremental lifetime cancer risk (ILCR) and safe monthly consumption rate (CR_mm_) associated with the intake of these metal(loid)s are the main focus of this work.

## 2. Materials and Methods

### 2.1. Sampling

A total of 273 specimens of two species, shi drum *(Umbrina cirrosa)* and brown meagre (*Sciaena umbra*), from the Mediterranean Sea were purchased in fish markets of the Apulian region (south Italy) during May–July 2020. A total of 147 specimens of shi drum (length range: 28.7–35.5 cm, average: 33.2 cm) and 126 specimens of brown meagre (length range: 22.7–26.0 cm, average: 24.3 cm) were gathered, according to species and size, into 5 pools. Specimens were dissected using scissors and stainless steel in order to avoid contamination. A pooled edible portion of similar-length specimens for each species was taken, homogenized and stored below −20 °C, pending analysis.

### 2.2. Chemical Analyses and Instrumental Conditions

The applied protocols and the instrumental conditions for measuring metal concentrations have been described in detail elsewhere [14]. Briefly, aliquots (about 1.0–2.0 g) of the samples were digested to a transparent solution with a mixture of HNO_3_-HClO_4_ (8:3) for Cd and Pb determination and with a mixture of H_2_SO_4_–HNO_3_ (1:1) for Hg and As. The completely digested samples were allowed to cool and were diluted with deionized water according to the method recommended by the official Italian agencies [20]. The metals content was determined by an atomic absorption spectrophotometer (Shimadzu AA 7000, Milan, Italy). Cd and Pb were analyzed using a graphite furnace (high-density tube) (GFA-7000), whereas Hg and As were analyzed using a hydride vapor generator (HVG-1) after reduction by NaBH_4_.

### 2.3. Quality Control and Assurance

Reference tissue (Tort-2 Lobster Hepatopancreas, National Research Council of Canada, Ottawa, ON, Canada) was treated and analyzed in the same way as the samples. Results (Hg: 0.28 ± 0.03; Cd: 25.8 ± 1.4; Pb: 0.32 ± 0.18; As: 20.5 ± 2.0 µg g^−1^ dry weight) were in agreement with the certified values (Hg: 0.27 ± 0.06; Cd: 26.7 ± 0.6; Pb: 0.35 ± 0.13; As: 21.6 ± 1.8 µg g^−1^ dry weight), and the standard deviation was low (n = 3), proving the repeatability of the methods. The results for the standard reference material displayed recoveries of the elements ranging from 92 to 104% (n = 3). The limit of detection (LOD) (Hg: 5; Cd: 0.12; Pb: 10; As: 6 ng g^−1^ wet weight) is defined as the concentration corresponding to three times the standard deviation of blanks, and the limits of quantification (LOQs) are the following: Hg: 13; Cd: 0.40; Pb: 0.38; As: 18 ng g^−1^ wet weight. Two blank samples were analyzed together with each sample batch. Metal concentrations in blanks were below the detection limits in all the analyses. Blanks and calibration standard solutions were analyzed in a similar way to the digested sample solution, and calibration curves were constructed. Analyses were duplicated to check the reproducibility of the results. Relative standard deviations among replicates were always less than 10%. Recovery tests were performed for the investigated metals in selected samples by spiking analyzed samples with aliquots of the metal standards and then carrying out digestion. The recovery percentages ranged from 96 to 99%. Throughout the manuscript, metal concentrations are presented as µg g^−1^ wet weight basis.

### 2.4. Health Risk Assessment

The estimated intake, target hazard quotient, incremental lifetime cancer risk (for Hg, the value of the cancer slope factor is not available, and consequently, carcinogenic was not calculated) and estimation of safe monthly consumption rate were calculated for two different scenarios of fish consumption (general population (GP): ingestion rate of 38.8 g day^−1^; high-level fish consumers (HC): ingestion rate of 71.0 g day^−1^) [21]. The target hazard quotient, the incremental lifetime cancer risk and the consumption limit calculations for Hg and As were performed assuming that Hg measured in fish was integrally in its methylated form (MeHg, 100% of the total Hg) and taking into account that inorganic As (iAs, 10% of the total As) in fish muscle represents a small portion of the total As content [22].

#### 2.4.1. Estimated Intake

The estimated intake (EI) (μg kg^−1^) was calculated utilizing the following formula:

EI = C × IR/BW

where C = chemical concentration in marine organisms (μg g^−1^ wet weight), IR = ingestion rate (g day^−1^) and BW is the average body weight (69.7 kg) [21] of the general population. The estimated weekly intakes were compared with the provisional tolerable weekly intake (PTWI) for Hg (4 µg kg^−1^ bw week^−1^ [23]), MeHg (1.6 µg kg^−1^ bw week^−1^ [24]) and Pb (25 µg kg^−1^ bw week^−^^1^ [25]) as well as with provisional tolerable monthly intake (PTMI) for Cd (25 µg kg^−1^ bw month^−1^ [26]). For inorganic As (iAs), the range of benchmark dose lower confidence limit (BMDL_01_) values for 1% excess risk of cancers of the lung, skin and bladder, as well as skin lesions, was 0.3–8 μg kg^−1^ bw day^−1^ [27].

#### 2.4.2. Target Hazard Quotient and Hazard Index

The target hazard quotient (THQ), which is a numeric estimate of the systemic toxicity potential posed by a single element within a single route of exposure, is described by the following equation [28]:

THQ = EFr × ED × FIR × C/RfD × WAB × AT × 10^−3^

where EFr = exposure frequency (365 days/year); ED = exposure duration (70 years), equivalent to the average lifetime in Italy; FIR = food ingestion rate (g day^−1^); C = metal concentration (mg kg^−1^ w w); RfD = oral reference dose (in mg kg^−1^ day^−1^): methylmercury (MeHg) = 1.0 × 10^−4^ mg kg^−1^ day^−1^, Cd = 1.0 × 10**^−4^** mg kg^−1^ day^−1^, Pb = 3.6 × 10^−2^ mg kg^−1^ day^−1^, inorganic arsenic (iAs) = 3 × 10^−4^ mg kg^−1^ day^−1^ [29,30]; and WAB = average body weight (kg); AT = averaging exposure time for non-carcinogens (365 days/year × ED). Hazard Index (HI) determines the potential risk triggered by metals collectively and is calculated as sum of THQ [28].

HI = THQ_Hg_ + THQ_Cd_ + … THQ_n_ …

If the HI value is under “1”, an adverse effect is out of the question in terms of human health.

#### 2.4.3. Incremental Lifetime Cancer Risk

The incremental lifetime cancer risk (ILCR), which constitutes a conservative estimate of the incremental probability that an individual will develop cancer during one’s lifetime as a result of a specific exposure to a carcinogenic compound [31], was estimated using the following equation [28]:

ILCR = CDI × CSF

where CSF = cancer slope factor of the elements considered (Cd = 15 mg kg^−1^ day^−1^, Pb = 8.5 × 10^−3^ mg kg^−1^ day^−1^, iAs = 1.5 mg kg^−1^ day^−1^ [30,32]) and CDI = chronic daily intake of chemicals (mg kg^−1^ bw day^−1^) represents the lifetime average daily dose of exposure to the chemical and was calculated through the following equation [28]:

CDI = (EDI × EFr × EDtot)/AT

where EDI = estimated daily intake; EFr = exposure frequency (365 days/year); EDtot = exposure duration (70 years), equivalent to the average lifetime; AT = averaging exposure time for non-carcinogens (365 days/year × ED). The US EPA [31] cancer risk considered de minimus or acceptable for regulatory purposes is within the range of 1 × 10^−6^ to 1 × 10^−4^. The cumulative cancer risk as a result of exposure to multiple carcinogenic heavy metals was assumed to be the sum of the individual metal(loid) increment risks.

#### 2.4.4. Estimation of Safe Monthly Consumption Rate

The safe monthly consumption rates that could be consumed over a month and would not be expected to cause any chronic systemic effects (CR_mm_) were evaluated for Hg, Cd, Pb and As according to the US EPA [29]. The following equation was used to calculate the allowable daily fish consumption rate (CR_lim_) (g day^−1^) [29]:

CR_lim_ = (RfD × BW) / C

where RfD is the reference dose (MeHg: 1 × 10^−4^ mg kg^−1^ day^−1^, Cd: 1.0 × 10**^−4^** mg kg^−1^ day^−1^, Pb: 3.6 × 10^−2^ mg kg^−1^ day^−1^, iAs: 3 × 10^−4^ mg kg^−1^ day^−1^) determined by the US EPA [30], BW is the average body weight of the consumer (69.7 kg) and C is the measured concentration of Hg, Cd, Pb and As in the edible portion of a given species of fish (µg g^−1^). The safe monthly consumption rate (CR_mm_) (meals/month) was calculated according to the following formula:

CR_mm_ = (CR_lim_ × Tap) / MS

where Tap is the average exposure time (30.44 days per month) and MS is meal size (0.227 kg fish/meal for adults) [29].

### 2.5. Statistical Analysis

The non-parametric Kruskal–Wallis test was used to test the hypothesis about concentration differences as a function of fish species and to determine whether there were differences in the levels of metal(loid) accumulation. A simple regression analysis was used to examine the correlations between the metal(loid) load and the length of fish samples. The length was considered the basic measure because it is less likely to be subject to major fluctuations than weight [33]. The level of significance was set at *p* ≤ 0.05. All statistical analyses were performed using XLSTAT-R version 2019.1 (Addinsoft, Paris, France).

## 3. Results and Discussion

### 3.1. Metal(loid) Levels and Accumulation Versus Fish Length

The descriptive statistics (range, average and standard deviation) of four metal(loid)s determined in the muscle tissue of two fish species are listed in Table 1. The average level sequence of the tested elements for both species was in the following order: As > Hg > Pb > Cd. The average concentrations of As, Hg, Pb and Cd were as follows: 0.59, 0.18, 0.10 and 0.07 μg g^−1^ w.w. in shi drum and 0.69, 0.19, 0.12 and 0.10 μg g^−1^ w.w. in brown meagre, respectively. According to statistical results, there was no significant difference in Hg concentrations between the two fish species examined (*p* > 0.05).

This concentration homogeneity is suggestive of the fact that the two fish species share a similar trophic niche (shi drum: 3.51; brown meagre: 3.80) [39,40]. Both are demersal species inhabiting various types of sea bottoms or rocky or sandy substrates, where they feed on a wide variety of organisms. Invertebrates and fish are the most important prey species in the diet of shi drum [41], whereas brown meagre generally feed on crustaceans, with an evident preference for decapods, although they become increasingly piscivorous as they grow [42]. This size-related shift in food preferences offers a chance to explore intraspecific differences in metal(loid) concentrations, above all, with regard to Hg. It is well known that a piscivorous diet predisposes to a higher accumulation of Hg, attesting the biomagnification process of this toxic metal through the entire food web. Several studies have shown the tendency of Hg to increase over time during the growth of fish as result of the continual accumulation and a slow depuration process of Hg by fish tissues [43]. Consistent with these assumptions, the results of linear regression analysis revealed a positive Hg-level–body-length relationship in both species. Such a correlation was highlighted by a more pronounced regression slope in brown meagre, with a *p* value equal to the limit of significance (*r* = 0.88, *p* = 0.05), relative to shi drum, where the increase was weaker and not significant (*r* = 0.69, *p* = 0.20). The stronger correlation noted in brown meagre compared to shi drum might be linked to the change in its feeding strategy, which leads older fish to a piscivorous diet, which is central for Hg bioaccumulation. Concerning Cd, the concentrations did not vary greatly between the two studied species (*p* > 0.05), but accumulation versus fish length revealed a different scenario. A significant decrease in Cd concentrations with increasing fish length (*r* = −0.91, *p* = 0.03) was noted in brown meagre, whereas there was not a well-defined relationship in shi drum (*r* = 0.42, *p* > 0.05), presumably as a result of the effect of the narrow concentration range detected in this species. The relationships between Cd levels and fish morphometric variables have been widely explored and generally described as negative [44,45]. With regard to the slope trend in brown meagre, the higher Cd concentrations in smaller fish classes than those of mature fish might be, once again, related to a shift in feeding habits, whereby juvenile fish consume a diet that includes a large percentage of crustaceans, which appear to retain high concentrations of this metal [46]. In case of Pb, the levels in muscle tissue of the two considered fish species differed little (*p* > 0.05). Concentrations within each species also remained rather constant, although increased element enrichment was observed in the muscle of medium-length fish. This moderate variability in concentrations resulted in no relationship between metal accumulation and fish length in either species (shi drum: *r* = −0.44, *p* = 0.46; brown meagre: *r* = −0.47, *p* = 0.42). However, it must be remembered that in the recent decades, corrective actions aimed at reducing Pb emissions in the environment have been carried out. This has resulted in a decrease in dissolved Pb content in coastal seawater of about 50% [47], so the concentrations of this element in fish are often very low. For As, no appreciable concentration difference was noted between the two species (*p >* 0.05), although slightly lower levels were detected in shi drum (0.59 μg g^−1^ w.w.) as compared with brown meagre (0.69 μg g^−1^ w.w.). Furthermore, the results of linear regression analysis highlighted the lack of a concentration–length relationships for both species (shi drum *r* = 0.23, *p* > 0.05; brown meagre *r* = 0.49, *p* > 0.05). Conflicting results have been reported concerning the presence of such correlations in fish. For example, Vieira et al. [48] reported a significant negative correlation between As concentrations and body length in various mackerel species. A positive but not significant correlation was observed in a Mediterranean scorpaenid [49], whereas a significant positive trend was encountered in different fish species from the western Mediterranean Sea [50]. Existing studies have shown that the dietary habits of marine organisms have a major influence on As levels. For example, fish that feed on organisms in a macroalgae-based food chain generally have high levels of As because algae contain considerable concentrations of this metalloid [27]. Consequently, marine organisms feeding on crustaceans have a consistently higher level of As than piscivorous species [51]. However, if nutritional preference is the only factor that induces the load of this element in ichthyofauna, we should have found a significant positive correlation at least in shi drum, which feeds mainly on crustaceans. The non-existence of such a relationship highlights that bioaccumulation of metal(loid)s within an organism is a process resulting from complex interactions between physiological (growth, weight loss, absorption and accumulation), chemical (metal concentration, speciation, bioavailability and pH) and environmental factors (temperature and food concentration), which in turn influence the absorption, metabolism and excretion of metals by the marine biota [52].

### 3.2. Comparison with Literature Data

As stated above, the metal load in aquatic inhabitants is affected by a series of intricate relations between intrinsic and extrinsic factors. Consequently, when evaluating the concentrations of elements in fish, it is more interesting to compare the results of the same species with studies performed in the same water body. To our knowledge, no studies are currently available regarding the concentrations of metals in these fish from the Italian coasts. Furthermore, analyses of trace metals in muscle tissue of these species in other geographic regions are also scarce. However, in general, Hg concentrations in shi drum are comparable with those recorded in the same species from an unpolluted area of the Mediterranean Sea (Abo-Kamash area, Libya) (0.22 μg g^−1^ w.w.) [53] but lower than those measured in brown meagre of Farwa Island (Mediterranean Sea, Libya) (0.34 μg g^−1^ w.w.), which is considered a highly sensitive area because of anthropogenic activities [54]. Concerning Cd, the concentrations in the edible tissue of the studied species are slightly higher than the values reported in the literature for shi drum in a low-contamination marine area located on the Sinop coast of the Black Sea (Turkey) (0.041–0.047 μg g^−1^ w.w.) [55] but lower than those reported for brown meagre in the North Aegean Sea (Turkey) (0.13 μg g^−1^ w.w.) [56]. The values of Pb are lower than those measured in shi drum from the Black Sea (0.20–0.22 μg g^−1^ w.w.) [55] but comparable with the levels described for brown meagre from Izmir Outer Bay (Mediterranean Sea) (0.154 μg g^−1^ w.w.) [57]. For As, data relative to Mediterranean Sea region are lacking, although an overview of studies performed in other water bodies showed that values registered here fall within the range reported for other fish belonging to the Sciaenidae family (0.48–1.00 μg g^−1^ w.w.) [58,59,60].

### 3.3. Health Risk Assessment

Multiple approaches have been used to assess the human health risks from fish consumption: (1) comparisons of metal(loid) concentrations with the maximum permissible limits (MPLs) for fish set by European and international standards; (2) comparison of estimated intake (EI) values of elements with the established provisional tolerable weekly/monthly intake (PTWI/PTMI) and with the range of benchmark dose lower confidence limit (BMDL_01_ = 0.3–8 μg kg^−1^ bw day^−1^) values for inorganic arsenic; (3) estimation of target hazard quotient (THQ) and hazard index (HI) for non-carcinogenic effects; (4) estimation of incremental lifetime cancer risk (ILCR) for carcinogenic effects; and (5) determination of the number of allowable fish meals per month (CR_mm_) to reduce the risk of chronic systemic effects.

#### 3.3.1. Metal(loid) Concentrations vs. International Dietary Standards

The European Union (EU) has set MPLs for Hg (0.5 μg g^−1^ w.w.), Cd (0.05 μg g^−1^ w.w.) and Pb (0.30 μg g^−1^ w.w.) in fish [34,35,36] (Table 1). According to the current regulations, Hg and Pb levels were below the established threshold values, whereas the concentrations of Cd exceed the MPL in all samples, with an only exception for the samples of smaller shi drum. This means that according to EU legislation, these fish are not legal to put on the market. For As in fish, no MPL has yet been established by the EU. However, some countries have stated a maximum limit for this metalloid in marine products. For example, in Australia and New Zealand, the maximum limit is 2 μg g^−1^ w.w. for fish and crustacea, [38], whereas the Brazilian guideline [37] allows a maximum permissible limit of As concentration in fish of 1 μg g^−1^ w.w. With reference to these international standards, none of the fish species sampled in this study exceeded the MPLs.

#### 3.3.2. Estimated Intake

Concentrations below MPLs are an important first step in providing the best possible quality of food, thus protecting public health. However, it is worth emphasizing that a food that does not exceed the maximum food standard does not necessarily mean that it is suitable for human consumption, as the dose of a toxic metal that is obtained from food depends not only on the concentration but also on the frequency and the amount of food consumed. Taking this into account, it is important to integrate the concentrations detected in food with exposure assessment by constructing different food consumption scenarios (general population = 38.8 g day^−1^, high-level fish consumers = 71 g day^−1^). As can be seen in Table 2, the Hg, Cd and Pb dietary intake calculated for the investigated fish species for both general population (Hg shi drum: 0.69 μg kg^−1^ week^−1^, brown meagre: 0.74 μg kg^−1^ week^−1^, Cd shi drum: 1.10 μg kg^−1^ month^−1^, brown meagre: 1.64 μg kg^−1^ month^−1^, Pb shi drum: 0.40 μg kg^−1^ week^−1^, brown meagre: 0.46 μg kg^−1^ week^−1^) and the high-level fish consumers (Hg shi drum: 1.27 μg kg^−1^ week^−1^, brown meagre: 1.35 μg kg^−1^ week^−1^, Cd shi drum: 2.02 μg kg^−1^ month^−1^, brown meagre: 2.99 μg kg^−1^ month^−1^, Pb shi drum: 0.73 μg kg^−1^ week^−1^, brown meagre: 0.84 μg kg^−1^ week^−1^) were less than tolerable dietary intake limits, suggesting that consumption of these marine products can be considered safe (Table S1).

However, the toxicological profile of metals strongly varies depending on their chemical form. Key examples are As, where the inorganic species are comparatively more lethal and toxic than the organic forms; and Hg, where the organic form, methylmercury, (MeHg) is much more dangerous than inorganic mercury. As a consequence, an indirect and conservative approach to calculate the dietary exposure was adopted, assuming that Hg measured in fish was integrally in its methylated form. Concerning arsenic, the toxicological profile refers only to inorganic chemical species because the organic arsenic forms are relatively non-toxic for human health [61]. Consequently, according to the worst-case scenario established by the US EPA for the health risk assessment of As intake due to fish consumption [32], it was assumed that 10% of the As was in inorganic form. In this context, methylmercury exposure estimates were lower than the PTWI (MeHg: 1.6 μg kg^−1^ bw week^−1^), as stipulated by the Joint FAO/WHO Expert Committee on Food Additives [24]. Nevertheless, for consumers with a high fish intake, the exposure estimation (1.27−1.35 μg kg^−1^ bw week^−1^), accounting for more than 75% of the PTWI of the MeHg (PTWI%: 79.4–84.4%), was of concern. Regarding As, the EFSA considering the PTWI of 15 μg/kg bw week^−1^ established by the JECFA [62] inappropriate, indicates a range of benchmark dose confidence limit (BMDL_01_) value for inorganic As between 0.3 and 8 μg kg^−1^ bw day^−1^ [27]. According to the results of the present study, the estimated intakes of inorganic As (0.06–0.07 μg kg^−1^ bw day^−1^) being well below the lower part of the BMDL_01_ range would is not of concern for any consumer group.

#### 3.3.3. Non-Carcinogenic and Carcinogenic Risk Assessment

**Figure 1:**
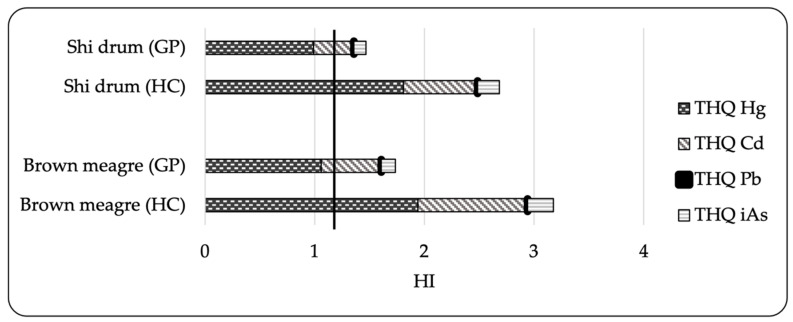
Target hazard quotient (THQ) and hazard index (HI) for the general population (GP) and high-level consumers (HC) through consumption of shi drum and brown meagre. Dark line represents the acceptable risk level of 1.

**Figure 2:**
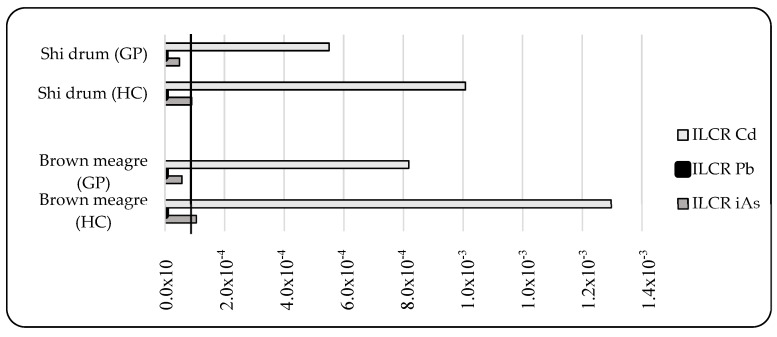
Incremental lifetime cancer risk (ILCR) for the general population (GP) and high-level consumers (HC) through consumption of shi drum and brown meagre. Dark line represents the threshold risk limit (ILCR > 10^−4^).

The non-carcinogenic target hazard quotient (THQ), hazard index (HI) and carcinogenic health risks (ILCR) are illustrated in Figure 1 and Figure 2 (Table S2). For the general population, none of the samples were observed to exceed the THQ threshold of Cd, Pb or As. The THQ values calculated for the population with a higher consumption scenario were also less than unity. However, Cd reaching the standard safe level of 1 for the consumption of brown meagre remains a point of concern for health risk. A more alarming picture emerged from THQ data for Hg, which was consistently close or above to 1 (general population: 0.99–1.06, high-level fish consumers: 1.81–1.94), indicating potential risk to humans due to the intake of this neurotoxin through the consumption of the two studied fish species. The values for the hazard index (HI), computed as the sum of THQ for individual elements, exceeded 1 in both consumption scenarios, which implies the exposed population may encounter non-carcinogenic health risks from the synergistic effect of the studied elements.

Many investigations have indicated that exposure to toxic elements increases the risk of cancer. Pb and MeHg are, in fact, classified as possibly carcinogenic to human, and Cd and As are known to cause a risk of cancer in humans [63]. According to the US Environmental Protection Agency (US EPA), lifetime health cancer risk (ILCR) values lower than 1 × 10^−6^ indicate that consumers are within the safe limit (the risk of developing cancer is 1 in 1,000,000); for values higher than 1 × 10^−4^, consumers are at risk (the risk of developing cancer is 1 in 10,000), whereas ILCR values higher than 1 × 10^−3^ signal that consumers are at considerable risk of developing cancer (the risk of developing cancer is 1 in 1000) [31]. As illustrated in Figure 2, the ILCR for Cd violated the threshold risk limit in all the studied fish; iAs only exceeded the risk in brown meagre, whereas none of the fish surpassed the designated risk limit for Pb in either consumption scenario. The cumulative risk indicates that general population was within the threshold risk of growing cancer (∑ILCR > 10^−4^), whereas the high-level fish consumers were at considerable cancer risk (∑ILCR > 10^−3^) through consumption of both species.

#### 3.3.4. Estimation of Safe Monthly Consumption Rate

To better assess and delineate the potential risks to human health, information related to the exposure assessment must be accompanied by an understanding of the amounts of fish that can be safely consumed over a given period of time without causing adverse effects. The US EPA [29] has recommended that the safe monthly consumption rate (CR_mm_ meals month^−1^) should be quantified to reduce the risk for fish consumers and elude chronic systemic effects. According to the US EPA [29], when the CR_mm_ of a meal is more than 16 meals per month, it is safe to consume. Following these guidelines, adults can consume more than 16 meals of these species based on the Pb and As concentrations. For Cd, the minimum and maximum number of fish meals allowed per month was 15 for shi drum and 10 for brown meagre, whereas according to concentrations of Hg, the number of meals allowed per month for both fish species was 6, which identifies a potential human health risk (Table S3).

## 4. Uncertainties and Limitations

Uncertainty is intrinsic in human health risk evaluation, even when the most precise data and the most refined models are applied. Consequently, when examining human exposure, uncertainties and limitations need to be listed, as they may limit the validity of the results presented. First, the lack of detailed consumption data relative to each tested fish species may lead to an uncertainty of the findings. Second, the contaminants were determined in fresh samples, and it has been demonstrated that culinary treatment leads to a change in element bioaccessibility, contributing to increased uncertainty of estimated exposure levels. Third, the As risk was calculated based not on a direct measure of the inorganic form but on an estimation of inorganic As from the total As in the fish muscle. Additional uncertainty may derive from the probability variables applied to estimate cancer risk. First of all, the ILCR for Hg was not estimated due the unavailability of the carcinogenic slope factor and the other carcinogenic slope factors, which were treated as a constant for all members of the population, can change depending on age group, as well as differences in dietary preferences, nutritional status and metabolism of individuals. However, even with these uncertainties, the results obtained in this study provide important information regarding the human health risk associated with metal(loid) exposure resulting from consumption of these fish species.

## 5. Conclusions

The present study provided baseline data on metal(loid) concentrations in two sciaenid species of high commercial significance, providing insight into the quality of these marine products and assessing the potential health risks for the general population and high-level fish consumers. Overall, the results revealed that the investigated fish species accumulated metal(loid)s in muscle tissue, with the highest concentrations of As, followed by Hg, Pb and Cd. Positive correlations between metal(loid) concentrations and fish length were observed exclusively for Hg, confirming that size and feeding of marine organisms play a crucial role in accumulation of this metal. Regarding human health, the average concentrations of metal(loid)s were below MPLs for human consumption designated by various health organizations, except for Cd. The estimated intakes for Hg, Cd and Pb calculated for both consumption scenarios were below the respective PTWI or PTMI, as well as those of inorganic As, which were even lower than the BMDL_01_. Non-carcinogenic risk assessment (THQ) did not reveal any concerns, except for Hg. The lifetime health cancer risk (ILCR) suggested hazard exclusively from Cd, although for high-level fish consumers, even the ILCR of As was, in some cases, above the acceptable range. Furthermore, the CR_mm_ calculated for Hg and Cd suggested a moderate consumption of these fish species, whereas there were no consumption limits based on the amount of Pb and As in fish tissue. In summary, the results of this study could be used as reference data for necessary future research, as the consumption of these species for a long period of time can lead to negative health effects due to the presence of metals such as Hg and Cd, especially for high-level fish consumers.

## Figures and Tables

**Table 1:** Metal(loid) concentrations (mg g^−1^ w.w.) and maximum permissible limits (MPLs) (μg g^−1^ w.w.) for human consumption.

	Length Range (cm)	N° Specimens		Hg	Cd	Pb	As
*Umbrina cirrosa* (Shi drum)	28.5–30.4	40		0.11 ± 0.03	0.05 ± 0.02	0.12 ± 0.03	0.58 ± 0.08
	30.5–32.4	36		0.18 ± 0.05	0.06 ± 0.02	0.09 ± 0.02	0.61 ± 0.09
	32.5–34.4	38		0.22 ± 0.04	0.09 ± 0.02	0.12 ± 0.03	0.49 ± 0.07
	34.5–36.4	23		0.18 ± 0.05	0.06 ± 0.02	0.08 ± 0.02	0.66 ± 0.10
	36.5–38.4	10		0.20 ± 0.05	0.07 ± 0.03	0.10 ± 0.03	0.60 ± 0.10
		Total 147	Min–Max Average ± St. Dev.	0.11–0.22 0.18 ± 0.04	0.05–0.09 0.07 ± 0.02	0.08–0.12 0.10 ± 0.02	0.49–0.66 0.59 ± 0.06
*Sciaena umbra* (Brown meagre)	20.5–22.4	32		0.14 ± 0.03	0.12 ± 0.03	0.12 ± 0.02	0.66 ± 0.09
	22.5–23.4	34		0.09 ± 0.02	0.13 ± 0.03	0.12 ± 0.03	0.58 ± 0.07
	23.5–24.4	23		0.18 ± 0.03	0.10 ± 0.03	0.16 ± 0.03	0.78 ± 0.11
	24.5–25.4	25		0.21 ± 0.04	0.07 ± 0.02	0.10 ± 0.03	0.61 ± 0.10
	25.5–26.4	12		0.33 ± 0.04	0.07 ± 0.02	0.09 ± 0.02	0.80 ± 0.12
		Total 126	Min-Max Average ± St. Dev.	0.09–0.33 0.19 ± 0.09	0.07–0.13 0.10 ± 0.03	0.09–0.16 0.12 ± 0.03	0.58–0.80 0.69 ± 0.10
MPLs				0.50 ^1^	0.05 ^1^	0.30 ^1^	1.0 ^2^, 2.0 ^3^

^1^ [34,35,36]; ^2^ [37]; ^3^ [38].

**Table 2:** Estimated exposure to metal(loid)s (μg kg^−1^ week^−1^ for Hg and Pb, μg kg^−1^ month^−1^ for Cd and μg kg^−1^ bw day^−1^ for iAs) in the different scenarios proposed (general population (GP): ingestion rate, 38.8 g day^−1^; high-level fish consumers (HC): ingestion rate, 71.0 g day^−1^).

	EWI_Hg_		PTMI_Cd_		EWI_Pb_		EDI_iAs_	
	GP	HC	GP	HC	GP	HC	GP	HC
*Umbrina cirrosa* (Shi drum)	0.43–0.86	0.78–1.57	0.84–1.50	1.53–2.75	0.31–0.47	0.57–0.86	0.03–0.04	0.05–0.07
	0.69 ± 0.16	1.27 ± 0.30	1.10 ± 0.25	2.02 ± 0.46	0.40 ± 0.07	0.73 ± 0.13	0.03 ± 0.003	0.06 ± 0.01
*Sciaena umbra* (Brown meagre)	0.35–1.29	0.64–2.35	1.17–2.17	2.14–3.97	0.35–0.62	0.64–1.14	0.03–0.04	0.06–0.08
	0.74 ± 0.35	1.35 ± 0.64	1.64 ± 0.46	2.99 ± 0.85	0.46 ± 0.10	0.84 ± 0.19	0.04 ± 0.01	0.07 ± 0.01
PTWI/PTMI	Hg ^1^:4; MeHg ^2^:1.6		25		25		-	
BMDL_01_	-		-		-		0.3–8	

PTWI = provisional tolerable weekly intake; PTMI = provisional tolerable monthly intake; BMDL_01_ = benchmark dose lower confidence limit; ^1^ [23]; ^2^ [24].

## Supplementary Materials

The following supporting information can be downloaded at: https://www.mdpi.com/article/10.3390/toxics10050223/s1, Table S1: Estimated daily (μg kg^−1^ bw day^−1^), weekly (µg kg^−1^ bw week^−1^) and monthly (µg kg^−1^ bw month^−1^) intakes for the general population (GP) and high-level consumers (HC) through consumption of shi drum and brown meagre; Table S2: target hazard quotient (THQ) and hazard index (HI) for the general population (GP) and high-level consumers (HC) through consumption of shi drum and brown meagre in different length ranges; Table S3: daily (CR_lim_: g day^−1^) and monthly (CR_mm_: meals month^−1^) consumption rate limit.
