# Supplementary material for: Residual Levels of Mercury, Cadmium, Lead and Arsenic in Some Commercially Key Species from Italian Coasts (Adriatic Sea): Focus on Human Health
Source: Toxics. 2022 Apr 28;10(5):223. doi: 10.3390/toxics10050223 (PMC9144595; doi:10.3390/toxics10050223)
Supplement: Supplementary file 1 [file toxics-10-00223-s001.zip › toxics-1682739-supplementary.pdf]

toxics

MDPI

Supplementary Materials: Residual Levels of Mercury, Cadmium, Lead and Arsenic in Some Commercially Key Species from Italian Coasts (Adriatic Sea): Focus on Human Health

Grazia Barone, Arianna Storelli, Rita Garofalo, Rosanna Mallamaci and Maria Maddalena Storelli

Table S1. Estimated daily (µg kg^{-1} bw day^{-1}), weekly (µg kg^{-1} bw week^{-1}) and monthly (µg kg^{-1} bw month^{-1}) intakes for the general population (GP) and high-level consumers (HC) through consumption of shi drum and brown meagre.

	Length range (cm)	EWI_{Hg}		$PTMI_{Cd}$		EWI_{Pb}		EDI_{iAs}	
		GP	HC	GP	HC	GP	HC	GP	HC
Umbrina cirrosa	28.5–30.4	0.43	0.78	0.84	1.53	0.47	0.86	0.03	0.06
(Shi drum)	30.5–32.4	0.70	1.28	1.00	1.83	0.35	0.64	0.03	0.06
	32.5–34.4	0.86	1.57	1.50	2.75	0.47	0.86	0.03	0.05
	34.5–36.4	0.70	1.28	1.00	1.83	0.31	0.57	0.04	0.07
	36.5–38.4	0.78	1.43	1.17	2.14	0.39	0.71	0.03	0.06
Min–Max		0.43–0.86	0.78–1.57	0.84–1.50	1.53–2.75	0.31–0.47	0.57–0.86	0.03–0.04	0.05–0.07
Average ± St. Dev.		0.69 ± 0.16	1.27 ± 0.30	1.10 ± 0.25	2.02 ± 0.46	0.40 ± 0.07	0.73 ± 0.13	0.03 ± 0.003	0.06 ± 0.01
Sciaena umbra	20.5–22.4	0.55	1.00	2.00	3.67	0.47	0.86	0.04	0.07
(Brown meagre)	22.5–23.4	0.35	0.64	2.17	3.97	0.47	0.86	0.03	0.06
	23.5–24.4	0.70	1.28	1.67	3.06	0.62	1.14	0.04	0.08
	24.5–25.4	0.82	1.50	1.17	2.14	0.39	0.71	0.03	0.06
	25.5–26.4	1.29	2.35	1.17	2.14	0.35	0.64	0.04	0.08
Min–Max		0.35–1.29	0.64–2.35	1.17–2.17	2.14–3.97	0.35–0.62	0.64–1.14	0.03–0.04	0.06–0.08
Average ± St. Dev.		0.74 ± 0.35	1.35 ± 0.64	1.64 ± 0.46	2.99 ± 0.85	0.46 ± 0.10	0.84 ± 0.19	0.04 ± 0.01	0.07 ± 0.01

Ingestion rate (GP = 38.8 g day^{-1}, HC = 71 g day^{-1}); PTWI = Provisional Tolerable Weekly Intake; PTMI = Provisional Tolerable Monthly Intake.

toxics MDPI

Table S2. Target Hazard Quotient (THQ) and Hazard Index (HI) for the general population (GP) and high-level consumers (HC) through consumption of shi drum and brown meagre in different length ranges.

	Length range (cm)	THQ_{Hg}		THQ_{Cd}		THQ_{Pb}		THQ_{iAs}		HI	
		GP	HC	GP	HC	GP	HC	GP	HC	GP	HC
Umbrina cirrosa	28.5–30.4	0.61	1.12	0.28	0.51	0.002	0.003	0.11	0.20	1.00	1.83
(Shi drum)	30.5–32.4	1.00	1.83	0.33	0.61	0.001	0.003	0.11	0.21	1.45	2.65
	32.5–34.4	1.22	2.24	0.50	0.92	0.002	0.003	0.09	0.17	1.82	3.33
	34.5–36.4	1.00	1.83	0.33	0.61	0.001	0.002	0.12	0.22	1.46	2.67
	36.5–38.4	1.11	2.04	0.39	0.71	0.002	0.003	0.11	0.20	1.62	2.96
Min–Max		0.61–1.22	1.12–2.24	0.28–0.50	0.51–0.92	0.001–0.002	0.002–0.003	0.09–0.12	0.17–0.22	1.00–1.82	1.83–3.33
Average ± St. Dev.		0.99 ± 0.23	1.81 ± 0.42	0.37 ± 0.08	0.67 ± 0.15	0.002 ± 0.0003	0.003 ± 0.001	0.11 ± 0.01	0.20 ± 0.02	1.47 ± 0.30	2.69 ± 0.55
Sciaena umbra	20.5–22.4	0.78	1.43	0.67	1.22	0.002	0.003	0.12	0.22	1.57	2.88
(Brown meagre)	22.5–23.4	0.50	0.92	0.72	1.32	0.002	0.003	0.11	0.20	1.33	2.44
	23.5–24.4	1.00	1.83	0.56	1.02	0.002	0.005	0.14	0.26	1.71	3.12
	24.5–25.4	1.17	2.14	0.39	0.71	0.002	0.003	0.11	0.21	1.67	3.06
	25.5–26.4	1.84	3.36	0.39	0.71	0.001	0.003	0.15	0.27	2.38	4.35
Min–Max		0.50–1.84	0.92–3.36	0.39–0.72	0.71–1.32	0.001–0.002	0.003–0.005	0.11–0.15	0.20–0.27	1.33–2.38	2.44–4.35
Average ± St. Dev.		1.06 ± 0.50	1.94 ± 0.92	0.55 ± 0.15	1.00 ± 0.28	0.002 ± 0.0004	0.003 ± 0.001	0.13 ± 0.02	0.23 ± 0.03	1.73 ± 0.39	3.17 ± 0.71

Table S3. Daily (CR_{lim}: g day^{-1}) and monthly (CR_{mm}: meals month^{-1}) consumption rate limit.

	**Length range (cm)**	**Hg**		**Cd**		**iAs**	
		**CR_{lim}**	**CR_{mm}**	**CR_{lim}**	**CR_{mm}**	**CR_{lim}**	**CR_{mm}**
Umbrina cirrosa	28.5–30.4	64	9	140	19	362	49
(Shi drum)	30.5–32.4	39	5	117	16	344	46
	32.5–34.4	32	4	78	10	429	57
	34.5–36.4	39	5	117	16	318	43
	36.5–38.4	35	5	100	13	350	47
Min–Max		32–64	4–9	78–140	10–19	318–429	43–57
Average ± St. Dev.		42 ± 13	6 ± 2	110 ± 23	15 ± 3	361 ± 41	48 ± 6
Sciaena umbra	20.5–22.4	50	7	58	8	318	43
(Brown meagre)	22.5–23.4	78	10	54	7	362	49
	23.5–24.4	39	5	70	9	269	36
	24.5–25.4	33	4	100	13	344	46
	25.5–26.4	21	3	100	13	263	35
Min–Max		21–78	3–10	54–100	7–13	263–362	35–49
Average ± St. Dev.		44 ± 21	6 ± 3	76 ± 22	10 ± 3	331 ± 44	42 ± 6
